# Physical Activity, Exercise and Sport Programs as Effective Therapeutic Tools in Psychosocial Rehabilitation

**DOI:** 10.2174/1745017901814010006

**Published:** 2018-02-21

**Authors:** Federica Sancassiani, Sergio Machado, Antonio Preti

**Affiliations:** 1 Department of Medical Sciences and Public Health, University of Cagliari, Cagliari, Italy; 2 Center for Consultation-Liaison Psychiatry and Psychosomatics, University Hospital, University of Cagliari, Cagliari, Italy; 3 Laboratory of Panic and Respiration, Institute of Psychiatry of Federal University of Rio de Janeiro (IPUB/UFRJ), Rio de Janeiro, RJ, Brazil; 4 Physical Activity Neuroscience, Physical Activity Sciences Postgraduate Program - Salgado de Oliveira University, Niterói, Brazil

**Keywords:** Sport, Exercise, Physical activity, Psychosocial disability, Psychosocial rehabilitation, Multidisciplinary

## Abstract

People with severe psychosocial disabilities have a 20-years shorter lifespan due to chronic somatic comorbidities and the long-term consequences of the side-effects of antipsychotic drugs.

They often are sedentary and show lower levels of physical activity, factors which can contribute to their shorter lifespan, because of the greater cardiovascular risk.

An increasing amount of evidence, including clinical trials, pointed out that sport, physical activity and structured exercise programs improve physical and psychological wellbeing of people with psychosocial disabilities, playing also an important role against their social isolation and self-stigma.

The NICE and APA guidelines include exercise and physical activity for the management of depressive symptoms.

Safe and effective programs require multidisciplinary teams that should always include mental health professionals, able to recognize the psychosocial needs, the impact of symptomatology, the role of secondary effects of psychotropic medication, the effect of previous exercise history, the lack of motivation, the inexperience with effort intensity and the frustration of people with psychosocial disabilities.

According to the World Health Organization [1] mental, neurological and substance use disorders account for 13% of the total global burden of disease in the year 2004. Depression alone accounts for 4.3% of the global burden of disease and it is among the largest single causes of disability worldwide (11% of all years lived with disability globally), particularly for women”.

People with severe psychosocial disabilities (*i.e.* schizophrenia, major depressive and bipolar disorders) have a 20-years shorter lifespan due to chronic somatic comorbidities, such as diabetes, overweight, obesity, cardiovascular and dysmetabolic diseases, also including to the long-term consequences of the side-effects of antipsychotic drugs [2-4]. Persons with severe psychosocial disabilities are also significantly more sedentary and show lower levels of physical activity than healthy persons [4, 5], factors which can contribute to their shorter lifespan, because of the greater cardiovascular risk that these unhealthy habits entail. It must be emphasized that sedentary behavior and low physical activity levels are independent yet modifiable risk. It must be emphasized that sedentary behavior and low physical physical activity levels are independent yet modifiable risk factors for premature mortality of these people [4-6]. For this reason, the interest in the integration of sport, physical activity and exercise programs as a component of treatment and rehabilitation for this population has been growing over the last decades.

Over time, much evidence pointed out the benefits of sport, physical activity and structured exercise programs in the mental health promotion field [7-9], in particular for depressive and anxiety disorders [9-20].

Sport, exercise and physical activity are not synonymous [21, 22]. Traditionally, physical activity is referred to “any bodily movement produced by skeletal muscles that results in energy expenditure” [23] and its components are occupational, transport, domestic, and leisure time, which consists of exercise, sport, and unstructured recreation. From this perspective, most sports contribute to overall physical activity [24]. Exercise is a “planned, structured and repetitive bodily movement, the objective of which is to improve or maintain physical fitness” [23]. Sport is defined as “a subset of exercise that can be undertaken individually or as a part of a team. Participants adhere to a common set of rules or expectations, and a defined goal exists” [24].

Studies have shown that even a small increase in physical activity has a positive impact on symptoms, functioning, severity of the condition, physical health (*i.e.*: cardiovascular risk profile) and sleep quality in people with psychosocial disabilities [17, 25-31]. Recent evidence from clinical trials pointed out that these benefits include improvements about weigh, motor difficulties, psychiatric symptoms, cognitive and social functioning, self-esteem, self-efficacy and quality of life [32-42].

Furthermore, sport, exercise and physical activity programs seem to play an important role against social isolation [32-36], a typical phenomenon closely linked with the experience of suffering from a psychosocial disability [43].

Psychosocial disabilities cause an elevated burden in terms of lost opportunities and this leads to higher self-perceived stigma [44], as well as to lower self-efficacy and quality of life [45]. Conversely, when people with psychosocial disabilities are offered a chance of employing their time in engaging social activities, such as sport and exercise programs, from which they are often kept apart, they benefit from the opportunity [32-36].

The evidence available regarding the management of depressive symptoms has led to the inclusion of exercise and physical activity into the guidelines from the National Institute for Health & Clinical Excellence (NICE) [46] and the American Psychiatric Association (APA) [47]. The NICE guidelines [46] recommend regular physical activity programs, 3 times/week, 45-60 minutes over 12 weeks for people with persistent subthreshold depressive symptoms or mild-moderate depression. The APA guidelines [47] suggest that people with depression of any severity and without any comorbid medical contraindications in relation to physical activity and exercise should include them as an add-on treatment.

However, these guidelines do not include any specific recommendations about the intensity and the suitable dose for exercise and physical activity [48], as well as any considerations about administration (*i.e.*: which kind of health professionals should conduct these interventions). In so far there is consistent evidence that supervised aerobic exercise is effective in reducing depressive symptoms when carried out 3 times/week, at moderate intensity for at least 9 weeks [49]. It is unclear how the effects that were observed for mild to moderate depression are generalizable to more severe psychosocial disabilities. Overall, safe and effective programs are expected to require the involvement of multidisciplinary teams that should always include mental health professionals, able to recognize the psychosocial needs, the impact of symptomatology, the role of the secondary effects of psychotropic medication, the effects of previous exercise history, and the lack of motivation, inexperience with effort intensity, and the frustration of people with psychosocial disabilities [48].

The evidence on the potentially beneficial effects of physical activity, exercise and sports on the course of severe psychosocial disabilities, particularly as far their quality of life is concerned, make hopeful that in the future the care for people with psychosocial disabilities should always include a focus on improving their fitness. Adequate programs should be carried out in stimulating environments, by qualified healthcare professionals, able to motivate and support participants in maintaining an active lifestyle [50-52]. To achieve these goals, changes in the mental health care system [53, 54] and the recognition of the same priority to physical, social and mental health needs of people with psychosocial disabilities are required.
